# A review on development of placental transfusion in term and preterm infants

**DOI:** 10.3389/fped.2022.890988

**Published:** 2022-09-15

**Authors:** Jiangyi Lu, Guang Yue, Qianying Wang, Xiaofeng Zhou, Rong Ju

**Affiliations:** Neonatal Department, Chengdu Women’s and Children’s Central Hospital, School of Medicine, University of Electronic Science and Technology of China, Chengdu, China

**Keywords:** neonates, placental transfusion, delayed cord clamping, intact-umbilical cord milking, cut-umbilical cord milking

## Abstract

In recent years, it has been verified that placental transfusion can replenish blood volume of neonates, improve organ perfusion in the early postnatal stage, and facilitate the transition from fetal circulation to adult circulation. Meanwhile, placental transfusion can reduce the need for blood transfusion and the onset of intraventricular hemorrhage, necrotizing enterocolitis, bronchopulmonary dysplasia, and other complications. Furthermore, it can improve the iron store and the long-term prognosis of central nervous system, and reduce infant mortality. Different methods have been used, including delayed cord clamping, intact umbilical cord milking, and cut umbilical cord milking. The World Health Organization (WHO) and other academic organizations recommend the routine use of delayed cord clamping at birth for the most vigorous term and preterm neonates. However, details of placental transfusion should be clarified, and the short/long-term impacts of this technology on some infants with special conditions still require further study.

## Introduction

The concept of placental transfusion has evolved over a long period of time. Following delivery, the two umbilical arteries gradually contract and the bloodstream from the fetus to the placenta rapidly decreases. Meanwhile, the umbilical venous flow can be maintained for up to a few minutes (1, 2). Therefore, keeping the umbilical cord intact over a period of time, rather than clamping it immediately after birth, can increase blood volume for the neonate and facilitate the transition from fetal circulation to adult circulation. It also helps the perfusion of important organs. Perspectives on placental transfusion have rapidly expanded with the development of maternal-fetal medicine and neonatology, and now it has been accepted as a routine procedure at most deliveries. The World Health Organization (WHO), the American College of Obstetricians and Gynecologists, and the American Academy of Pediatrics all recommend delayed umbilical cord clamping (DCC) in most vigorous term and preterm infants at birth. However, there are still problems in special cases with regard to the benefits and risks of this technique, and further study is still needed (3, 4). This article seeks to review and summarize most current literature on placental transfusion. A table of studies evaluating the topic is presented (Table 1).

**TABLE 1:** Studies of placental transfusion in neonates.

First author, year of publication	Country	Study design	Characteristics of participants	Enrolled: (ECC/DCC/I-UCM/C-UCM)	Significant findings
McDonald and Middleton (14)	Australia	Meta-Analysis (11 RCT)	2989 mothers and their babies	ECC (<60 s) vs. DCC (>60 s)	• lDCC didn’t increase maternal postpartum hemorrhage. • lDCC increase jaundice in need of phototherapy. • lDCC increases hemoglobin levels.
Rabe et al. (15)	United Kingdom	Meta-Analysis (40 RCT)	4884 babies and their mothers. Babies were between 24 and 36^+6^ weeks’ gestation	ECC (<30 s) DCC (30–180 s) UCM (20 cm, 2–4 times milking) C-UCM	• lDCC vs. ECC: DCC probably reduces death before discharge; DCC may make little difference to IVH (grades 3 and 4); slightly reduces the any grade IVH; little or no difference in: PVL, CLD, maternal blood loss ≥ 500 mL. • lUCM vs. DCC/ECC: insufficient data for reliable conclusions.
Ortiz-Esquinas et al. (30)	Spain	Meta-Analysis (10 RCT)	1845 newborns	I-UCM (3–5 times) vs. ICC/DCC	• lI-UCM didn’t improve hematologic variables for newborns ≥ 34 weeks of gestation. • lI-UCM slight decrease in hemoglobin levels at 6 weeks when compare to DCC.
Fogarty et al. (34)	Australia	Meta-Analysis (18 RCT)	2834 infants born < 37 weeks’ gestation	ECC (<30 s) vs. DCC(≥30 s)	• lDCC reduced hospital mortality. • lDCC reduced the incidence of low Apgar score at 1 min, did not reduce intubation for resuscitation, admission temperature, mechanical ventilation, IVH, brain injury, CLD, PDA, NEC, late onset sepsis or retinopathy of prematurity. • lDCC increased peak hematocrit and reduced the proportion of blood transfusion by 10%. • lPotential harms of polycythemia and hyperbilirubinemia.
Al-Wassia and Shah (42)	Saudi Arabia	Meta-Analysis (7 RCT)	501 infants	I-UCM (2–5 times) vs. ICC/DCC/no intervention	For infants ≤ 33 weeks: • lI-UCM didn’t increase mortality, volume expanders or inotrope support. • lI-UCM increase levels of hemoglobin and hematocritin (first 48 h and 6 weeks of life). • lUCM reduced risk for oxygen requirement at 36 weeks’ PMA and IVH of all grades.
Nagano et al. (45)	Japan	Meta-Analysis (2 RCT)	255 preterm infants, 23 0/7 to 32 6/7 weeks of gestation	I-UCM (4 times) vs. DCC (≥30 s)	• lI-UCM reduce IVH and increased Bayley score at 2 years of age compared to DCC.
Balasubramanian et al. (46)	India	Meta-Analysis (19 RCT)	2014 preterm infants	(1) ()I-UCM vs. DCC (2) ()I-UCM vs. ICC (3) ()C-UCM vs. ICC/DCC	• lCompared to DCC, I-UCM increased IVH (grade 3 or 4). • lCompared to ICC, I-UCM reduced the need for packed RBC transfusions.
Chen et al. (22)	China	RCT	720 term mothers/infants	ECC (<15 s, *n* = 90) vs. DCC (by 30, 60, 90, 120, 150, and 180 s, or when the cord pulsation ceased, *n* = 90 in each group)	• lDCC for 30 s didn’t improve the mean hematocrit levels at 24 h after delivery when compare with ICC. • lDCC for 30–180 s didn’t change the neonatal and maternal outcomes (hyperbilirubinemia or phototherapy, mother postpartum blood-loss).
Andersson et al. (23)	Sweden	RCT	334 full term infants born after a low risk pregnancy	ECC (≤10 s, *n* = 166) vs. DCC (≥180 s, *n* = 168)	• lDCC had no superiority in hemoglobin levels at 4 months of age, but higher mean ferritin concentration and lower prevalence of iron deficiency.
Mercer et al. (24)	USA	RCT	73 term singleton infants, follow-up at 12 months of age	ECC (≤20 s, *n* = 166) vs. DCC (≥5 min, *n* = 168)	• lDCC increased myelin content in important brain regions involved in motor function, visual/spatial, and sensory processing at 12 months of age.
Andersson et al. (25)	Sweden	RCT	263 full-term infants born after a low-risk pregnancy	ECC (≤10 s, *n* = 141) vs. DCC (≥180 s, *n* = 122)	• lDCC improved scores in the fine-motor and social domains at 4 years of age, especially in boys.
Purisch et al. (31)	USA	RCT	113 women with scheduled cesarean delivery of term singleton gestations	ECC (≤15 s, *n* = 56) vs. DCC (60 s, *n* = 57)	• lDCC didn’t influence the maternal hemoglobin level at day 1.
Girish et al. (33)	India	RCT	101 infants (≥35 weeks) who were depressed at birth	ECC (*n* = 51) vs. I-UCM (20 cm, 3 times, *n* = 50)	• lNo differences in resuscitation delay, resuscitation efforts, and short-term outcomes (respiratory support, HIE, abnormal neurological examination, died, duration of hospital stay).
Katheria et al. (39)	USA	RCT	120 premature infants (23 0/7–31 6/7 weeks’ gestational age)	V-DCC (60 s, *n* = 63) vs. DCC (60 s, *n* = 62) (V-DCC: initial continuous positive airway pressure while DCC)	• lNo difference in peak hematocrit in the first 24 h of life and onset of breathing. • lDCC need a greater duration of stimulation than V-DCC. • lNo differences in delivery room interventions, early hemodynamics or neonatal outcomes.
Katheria et al. (43)	USA	RCT	60 infants < 32 weeks’ gestation	ICC (*n* = 30) vs. I-UCM (20 cm, 3 times, *n* = 30)	• lI-UCM had greater measures of superior vena cava flow and right ventricular output in the first 6 and 30 h of life, and greater serum hemoglobin, fewer blood transfusions, fewer days on oxygen therapy, and less frequent use of oxygen at 36 weeks’ PMA.
March et al. (44)	USA	RCT	75 infants between 24 and 28 weeks of gestation	ICC (*n* = 39) vs. I-UCM (20 cm, 3 times, *n* = 36)	• lI-UCM had higher hematocrits at birth, reduce transfusion need, lower the incidence of IVH.
Katheria et al. (47)	USA	RCT	197 infants < 32 weeks’ gestation	Cesarean delivery: DCC (45–60 s, *n* = 79) vs. I-UCM (4 times, *n* = 75); Vaginal delivery: DCC (*n* = 23) vs. I-UCM (*n* = 20)	• lCesarean delivery: I- –UCM had higher superior vena cava flow and right ventricular output in the first 12 h of life, and higher hemoglobin, delivery room temperature, blood pressure over the first 15 h, and urine output in the first 24 h of life. • lVaginal delivery: No differences.
Katheria et al. (48)	USA	RCT	540 preterm infants born at 23 0/7–31 6/7 weeks’ gestation	DCC (≥60 s, *n* = 238) vs. I-UCM (20 cm, 4 times, *n* = 236)	• lNo difference in death or severe IVH for infant < 32 weeks’ gestation. • lI-UCM increased the severe IVH among infants born at 23–27 weeks’ gestation compared to DCC.
Nevill and Meyer (38)	New Zealand	Observational study	124 infants ≤ 29 weeks	ICC (*n* = 62) vs. DCC (40 s, *n* = 62)	• lNo difference in 1 and 5 min Apgar scores, intubation at birth, admission temperatures. • lDCC increase CLD. • lThe non-breathing DCC infant was more likely to be intubated, have CLD, and severe IVH.
Takami et al. (40)	Japan	Observational study	50 VLBW infants < 29 weeks and birth weight < 1250 g	I-UCM (20 cm, 2–3 times, *n* = 26) vs. ICC (*n* = 24)	• lI-UCM had higher hematocrit, left ventricular end-diastolic dimension, left ventricular cardiac output, superior vena cava flow, and improved the LV Tei index, tissue oxygenation index and decreased cerebral fractional tissue oxygen extraction within 24 h after birth.
Kumbhat et al. (49)	USA	Observational study	1834 infants < 29 weeks of gestation	DCC (*n* = 1402) vs. I-UCM (*n* = 432)	• lI-UCM increased severe IVH by 36 weeks’ PMA. • lOther secondary outcomes were similar.
Simonin et al. (50)	USA	Observational Study	403 infants < 37 weeks gestation	C-UCM (20–30 cm, *n* = 106) vs. Controls (didn’t receive UCM, *n* = 297)	• lNo differences in hemoglobin/hematocrit, peak bilirubin values, the incidence of intraventricular hemorrhage, need for blood transfusions, and the use of pressors.
Qian et al. (21)	China	Retrospective study	1981 mother–infant pairs, healthy term infants	ECC (<30 s, *n* = 1009) vs. DCC (3 group: 30–60 s, 60–90 s, 90–120 s, *n* = 949)	• lDCC (<90 s) improve the early hematological status, didn’t increase jaundice requiring phototherapy. • lDCC for 90–120 s didn’t result in further increases in hemoglobin and hematocrit levels but led to a higher risk of polycythemia and jaundice requiring phototherapy.
Patel et al. (41)	USA	Retrospective cohort study	318 infants < 30 weeks of gestation	I-UCM (3 times, *n* = 158) vs. retrospective cohort (*n* = 160)	• lI-UCM improved hemodynamic stability, higher mean blood pressures through 24 h of age, and less vasopressor use. • lI-UCM increase initial hematocrit value and decrease red cell transfusions. • lI-UCM reduced IVH, NEC, death of hospitalization
El-Naggar et al. (51)	Canada	Comparative Study	9729 preterm infants < 33 weeks of gestation	ECC (<30 s, *n* = 4916), DCC (≥30 s, *n* = 4419),I-UCM (*n* = 394)	• lECC had higher mortality or major morbidity compared to UCM group. • lECC were associated with mortality and IVH when compared with DCC. • lUCM increased severe IVH when compared with DCC. • lRates of blood transfusion were higher with ECC compared with UCM and DCC, but no difference in peak serum bilirubin levels.
Hosono et al. (52)	Japan	Comparative Study	40 infants < 29 weeks of gestation	C-UCM (30 cm, *n* = 20) vs. I-UCM (20 cm, 2–3 times, *n* = 20)	• lNo difference in transfusion need during the hospital stay and the mean number of RBC transfusions given within the first 21 days of life.

ECC, early cord clamping; ICC, immediately cord clamping; DCC, delayed cord clamping; I-UCM, intact umbilical cord milking; C-UCM, cut umbilical cord milking; IVH, intraventricular hemorrhage; PVL, periventricular leukomalacia; PDA, patent ductus arteriosus; CLD, chronic lung disease; NEC, necrotizing enterocolitis; PMA, postmenstrual age.

## Physiological basis of placental transfusion

The placenta is an indispensable organ during pregnancy, which plays a variety of essential roles, such as providing a means of maternal-fetal oxygen and carbon dioxide exchange, supplying nutrients to the fetus, transporting various substances, secreting significant factors and hormones, and also acting as an immune organ and barrier to the fetus (5).

**FIGURE 1:**
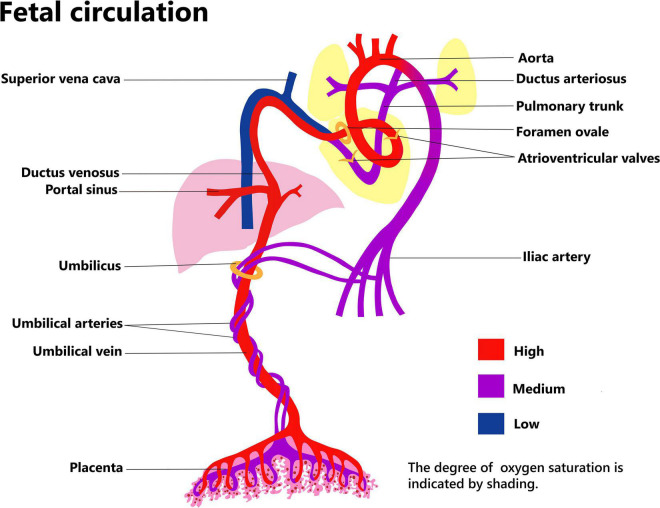
Fetal circulation.

Placental function relies on maternal and fetal circulation (Figure 1). The umbilical arteries originate from the bilateral internal iliac arteries, which pass through the umbilical ring in the fetal abdominal wall and enter the umbilical cord. Here, the two umbilical arteries and one umbilical vein form a rope-like structure that links the fetus and the placenta. Usually, the two umbilical arteries form an anastomotic connection at the site where the umbilical cord approaches the placenta, and then branch out into the placental villi. The umbilical vein passes through the umbilical ring and into the abdomen of the fetus. Some of the blood in the umbilical vein imports into the ductus venosus and enters the inferior vena cava, thereafter, passes from the right to the left atrium through the foramen ovale. Blood flowing into the left branch of the portal vein nourishes the left side of liver, while the remaining blood nourishes the right side of the liver, after passing through the ductus venosus (6).

The combined ventricular output (CVO) of the fetus was about 450 ml/min/kg over the latter two-thirds of gestation, the right ventricular output became the dominant at second half of second trimester (7). In the near-term fetus, around 15% of the CVO reaches the lungs, account for only a quarter of right ventricular output; the remaining three quarters blood flows through the ductus arteriosus to descending aortic. Around 75% relatively well-oxygenated left ventricular output perfuses the head, neck, and arms; the remaining 25% flows to the descending aorta. The less-oxygenated descending aorta blood flows to the abdomen, lower body and the placenta, of which placenta hold about 50% (30% of CVO) (8).

Umbilical cord blood flow increases steadily to about 105 ml/kg/min before 20 weeks of gestation, and then gradually decreases to about 65 ml/kg/min near term. Between 20 and 30 weeks of gestation, the placental circulation accounts for 30% of the fetus’s total cardiac output, and this drops to 20% near term. For fetuses with severe growth retardation, the blood flow to the placenta is only 10% or less. Most of the blood circulates within the fetus, however, this type of “self-circulation” without exchange with maternal substances would eventually lead to an exacerbation of fetal growth retardation due to lack of support and regulation from maternal sources via the placenta (1).

The placenta is the foremost blood storage site for the fetus during pregnancy. Compared with the fetus, it is relatively large, and during the second trimester it holds a similar blood volume to that of the fetal body, while near term it still holds about 1/3 of the fetal blood volume. The liver and other visceral organs have a limited buffering capacity for blood reserve, but they are inferior in this regard to the placenta (1, 9).

Placental blood delivery to the fetus is not only important during the prenatal period, but also during delivery. Here it can improve the blood volume of the newborn, which leads to a better coordination with the initiation of pulmonary circulation and the dramatic change of the systemic circulation load in the early postnatal period; these all help the neonate to make a smooth transition from fetal to adult circulation. With the support of placental transfusion, the perfusion of important organs is guaranteed in the immediate postnatal period, which contributes to reducing intraventricular hemorrhage (IVH) and necrotizing enterocolitis (NEC).

## The development of placental transfusion

Prior to the advancement of modern medicine, the common practice was to keep the umbilical cord intact until placental expulsion. There were even some reports of maintaining the umbilical cord attached to the placenta until natural separation, a practice termed “Lotus Birth”; however, this procedure increases the risk of neonatal sepsis (10–12).

From the 1940s–1950s, immediate cord clamping (ICC) after birth quickly became a common practice due to concerns on increasing maternal postpartum hemorrhage, although this practice was not supported by evidence (13). Actually, systematic retrospective analyses of DCC and ICC show that DCC doesn‘t increase the duration of the third stage of labor, maternal mortality, postpartum hemorrhage, nor the need for postpartum transfusion (14–16). Subsequently, two optional methods of umbilical cord milking (UCM) were developed: intact umbilical cord milking (I-UCM) and cut umbilical cord milking (C-UCM).

## Research and guidelines for placental transfusion in term infants

The effect of placental transfusion on neonates varies on conditions. Factors such as delayed ligation, lung inflation, mode of delivery, gravity, and the use of oxytocin to stimulate uterine contraction, can all impact the effect of placental transfusion (17–19).

Delayed umbilical cord clamping is the most important factor affecting placental transfusion. ICC is defined as clamping the umbilical cord within 10–15 s after birth. According to the 7th edition of the *Textbook of Neonatal Resuscitation*, DCC is recommended as the standard procedure at birth (2). The umbilical cord bloodstream should be maintained at least 30–60 s after birth for most vigorous term and preterm infants. Ideally, it should be maintained until umbilical cord pulsation stops. The Italian Guidelines for Placental Transfusion in 2018 made the same recommendation (14).

Delayed umbilical cord clamping plays a role in many ways, the continuous blood flow from the placenta to fetus replenishes blood volume in neonates, increases hemoglobin levels and hematocrit, and can also assist with maintenance of body temperature. Multiple studies have shown that increased hemoglobin level could effectively reduce the need for blood transfusion, without increasing the incidence of polycythemia and hyperbilirubinemia that require phototherapy (20–22). The benefits of DCC were even sustained into childhood. As described by Andersson et al. (23), infant performed DCC at least 3 min didn’t observed higher hemoglobin levels at 4 months of age compared with ICC (<10 s), but ferritin levels were superior than ICC group. Mercer et al. (24) studied a longer time of DCC, they compared more than 5-min delay in umbilical cord to ICC (≤20 s), found the similar results by Andersson et al. (23) moreover, greater myelin content in important brain regions were observed in DCC group when followed up to 12 months, and the brain regions involved in motor function, visual or spatial, and sensory processing. Another study by Andersson et al. (25) also reported the neurodevelopmental benefits from DCC in term infants, they found that infants receiving DCC (≥3 min) had better motor and social skills scores at age 4 than ICC (<10 s), especially in boys.

Neonates face enormous circulatory challenges after birth. Those who treated with ICC usually experience a sudden reduction in preload and a significant increase in afterload. Before birth, the unexpanded lung induces high pulmonary circulation resistance, with very little blood moving through the pulmonary circulation. Blood rich in oxygen and nutrients from the umbilical vein passes through the foramen ovale and systemic circulation, to mainly supply the brain and upper trunk. Since the resistance of placental circulation is low, blood flow in the umbilical arteries accounts for a large part of the systemic blood flow. A sharp increase of systemic circulatory resistance could be expected after birth due to immediate ligation of the umbilical cord and interruption of placental transfusion, which brings quick increase of left ventricular afterload. Thereafter, pulmonary circulation resistance decreases, and blood flow increases significantly when the lungs dilate; at this point blood flow through the foramen ovale into the systemic circulation falls quickly, and the preload of the left heart is presented with a transient and dramatic decrease. DCC can precisely supplement left ventricular blood volume and slowly improve systemic circulation resistance based on contraction of the umbilical arteries. Therefore, DCC can assist neonates to make a smoother transition from fetal circulation to adult circulation and maintain a more stable blood pressure (2, 13, 20, 26).

Due to the advantages far outweighing the disadvantages, DCC is now recommended for most vigorous term or preterm infants. Some researchers suggest that DCC should be extended to more neonates with special conditions. In congenital diaphragmatic hernia patients, organs such as the gastrointestinal tract herniate into the chest and cause a high incidence of persistent pulmonary hypertension, which has a great influence on the cardiorespiratory system (27, 28). These patients usually need advanced therapies. DCC is suggested to optimize the condition of infants with congenital diaphragmatic hernia during the early stage of birth, and to help them transit from fetal circulation to adult circulation. The Children’s Hospital of Philadelphia has begun an ongoing study of DCC in neonates with congenital diaphragmatic hernia. Similarly, infants with congenital heart disease often need respiratory support after birth, and sometimes even early surgical therapy. In recent years, researchers have noted that DCC did not increase the risk of asphyxia, or intubation in term infants with prenatally diagnosed congenital heart disease; it also reduced blood transfusion during hospitalization (29).

Many studies have shown that DCC has multiple advantages and it has been widely recognized, but the therapeutic effects are affected by many factors. In the third stage of labor, uterine contraction contributes to 25–30% of placental transfusion. The intrauterine umbilical venous pressure is about 40–50 mmHg, but it can reach 100 mmHg during a contraction. For neonates delivered by cesarean section, owing to the lack of increased umbilical venous pressure caused by uterine contraction, the effect of the opening of the pulmonary circulation and right ventricular afterload decreasing–that is caused by the newly established respiration–will play a greater role (9). Some scholars worry that the position of mother and baby will influence the effect of placental transfusion in DCC. A multi-center randomized controlled study published showed that the position of the neonate before cord clamping did not impact the effect of placental transfusion. When vigorous neonates born vaginally were held for 2 min before cord clamping at the level of the vagina or on the mother’s abdomen or chest, the volume of placental transfusion didn’t vary significantly (19). Based on the data, gravity seems to have little influence on DCC following a vaginal delivery. Current guidelines or specifications point out that putting those infants born vaginally on the mother’s abdomen after birth is also beneficial owing to the skin-to-skin contact that helps keep them warm. While neonates delivered by cesarean section do not experience effective uterine contraction, it is wise to put these babies below the mother’s position to promote the placental transfusion; at the same time, other measures should be taken to keep the baby warm. DCC and initial resuscitation should be performed simultaneously (2).

As a natural process, DCC does not increase the incidence of adverse reactions such as hyperbilirubinemia and polycythemia. However, DCC cannot be used in some term infants. Due to the need for emergent resuscitation, 14–22% of neonates undergo ICC. This is unfortunate because these infants may benefit the most from DCC (20). According to International Resuscitation Course, ventilation support is the most critical step during resuscitation (2). The 2012 WHO Guidelines for neonatal resuscitation suggested that DCC could be performed during positive pressure ventilation in hospitals where clinicians have the relevant experience, but this practice has not been recommended (23). It is also mentioned in the 2016 International Resuscitation course that the resuscitator should communicate with the obstetrician before delivery on the presence of placental abruption, placenta previa with bleeding, vasa previa with bleeding, or umbilical cord tearing, etc. It is believed that ICC should be adopted if the integrity of placental circulation is disturbed. Neonatologists need to engage in a lively discussion with obstetricians on some conditions, such as fetal retardation, ultrasound monitoring for abnormal umbilical blood flow, abnormal placenta, and other conditions that affect umbilical cord or placenta blood flow; DCC may be beneficial for neonates under such situations (2). Since most DCC studies excluded multiple pregnancies, there are no clear recommendation for newborns with multiple births. The 2018 Italian Guidelines for Placental Transfusion do not suggest DCC for single chorionic twins to avoid acute fetal transfusion syndrome; and the recommendation of DCC for dichorionic twins is also weak due to low level evidences. Shoulder dystocia, amniotic fluid embolism, feto-fetal transfusion syndrome, and mothers with HIV are all contraindications for DCC (13).

Given the above-mentioned issues with DCC, Umbilical cord milking (UCM) is another method of placental transfusion. For term infants, UCM has similar effects compared with DCC, and even superior to DCC in some respects (30, 31). The cord is milked only once in C-UCM, and the blood volume obtained by C-UCM may be less than that obtained by I-UCM (32). Generally, UCM takes less time than DCC, so I-UCM may shows advantages in asphyxiated newborns who needs immediate resuscitation. Small randomized controlled trial showed that, I-UCM used as a placental transfusion strategy in late preterm and term neonates requiring resuscitation with no significant adverse short-term outcomes compared to ICC. I-UCM maybe a feasible strategy for depressed infants, but still need more discussion, including long-term outcome study (33). UCM technology is mainly used in premature infants, whereas DCC is still preferred in term infant (10, 13, 26). UCM will be discussed more below.

## Advances in placental transfusion of premature infants

Premature infants are more likely to be admitted into NICU than term infants, some of them even need respiratory and circulatory support. Potentially they could benefit most from placental transfusion.

Delayed umbilical cord clamping plays an important role in premature infants. It can reduce in-hospital mortality, increase hematocrit, reduce the need for blood transfusion and inotropic, improve the mean arterial blood pressure in early hours after birth (14, 34). In recent years, a number of studies have found that intracranial hemorrhage in premature infants is closely related to insufficient blood flow in the superior vena cava. Compared with ICC, DCC can improve hemodynamic stability in early life and reduce the occurrence of intracranial hemorrhage (15, 35–37).

Although premature infants can also benefit from DCC, some studies show that DCC may be inadequate under some circumstances. Preterm deliveries often require more resuscitation, let alone this may happen with multiple pregnancy. DCC is also unfairly treated due to misunderstandings of the relationship between placental transfusion and maintenance of body temperature by some perinatal practitioners.

Aladangady et al. (17) found that the blood volume was significantly increased in vaginally born preterm infants (24–32 gestational weeks) receiving DCC procedure, but the same changes did not appear in premature infants delivered by cesarean section. Meanwhile, Nevill et al. (38) found that 70% of preterm infants (<29 gestational weeks) in the 40 s‘ DCC group established spontaneous breathing within 1 min after birth, and there was no significant difference in Apgar score, the number requiring intubation at birth, and admission temperatures compared with ICC group, Instead, the apneic infants in DCC group were more likely to be intubated, have chronic lung disease, and severe IVH. Characteristics of this subgroup led to a higher incidence of chronic lung disease in DCC group. Katheria et al. (39) found no significant difference on establishment of spontaneous breathing, cerebral oxygenation, cardiac output, and other neonatal outcomes in the two groups of preterm infants (<32 gestational weeks) initially stimulated or ventilated during DCC. This is the first successful exploration of feasibility of ventilatory support for preterm infants during DCC procedure. However, tactile stimulation didn’t show any advantages over positive pressure ventilation during DCC procedure, including neonatal mortality.

Meanwhile, the interest on UCM technique is also growing. For UCM, the cord remains intact after delivery. 20 cm‘ s umbilical cord is held within the thumb and forefinger and gently squeezed 2–4 times, the blood will be forced to flow from the cord to the neonate at a rate about 10 cm/s (9, 13).

The benefits of UCM over ICC have been widely verified. UCM can significantly improve the clinical status of preterm babies, such as blood volume, hemoglobin level, arterial pressure, cerebral oxygenation, urine output during early life, and effectively reduce blood transfusion (40–43). A meta-analysis showed that UCM had no significant effect on hospital mortality, NEC, chronic lung disease (CLD), sepsis, respiratory distress syndrome (RDS), patent ductus arteriosus (PDA) and retinopathy of prematurity (ROP) compared with ICC (15). Hemodynamics evaluation by ultrasound also shows that premature infants treated by UCM were superior to ICC in left ventricular end-diastolic volume, left ventricular cardiac output, and superior vena cava blood flow (41). Regarding the nervous system, several studies have suggested that UCM can effectively reduce the occurrence of preterm IVH compared with ICC (42–44).

Previous studies have suggested that UCM may be superior to DCC in preterm infants. Meta-analysis by Nagano et al. (45) demonstrated low quality evidence for reduced IVH and improved Bayley score at 2 years of age with UCM compared to DCC in preterm infants; UCM (4 stripping) provided more blood volume than DCC (30–60 s) in this study, but longer duration of DCC haven’t been investigated. Recent studies warning that UCM may be harmful to premature babies. Systematic reviews by Balasubramanian et al. (46) comparing UCM with DCC in preterm infants have reported that UCM significantly increased the risk of severe intraventricular hemorrhage in preterm infants < 34 weeks of gestation; this result were mainly influenced by the large multi-center trial conducted by Katheria et al. (48). Retrospectively data from Canadian Neonatal Network showed that both DCC and UCM were associated with better short-term outcomes than ICC in preterm infants; however, the odds of severe intraventricular hemorrhage were higher with UCM compared with DCC both in gestational age < 33 weeks and < 28 weeks (51). Result from another multicenter retrospective study also reported that extremely preterm infant exposed to UCM had higher odds of severe IVH than DCC (49).

Besides, another feasible method is C-UCM, which has been initially explored in some Asian countries. The cord was cutted after delivery, remained a 30–40 cm‘ s segment attach to newborn. The cutted cord was gently squeezed once at a rate about 10 cm/s. Hosono et al. (52) verified the safety and effectiveness of the C-UCM technique, and found that there was no significant difference in blood transfusion requirements between C-UCM and I-UCM group. Blood volume transported to the baby was limited by squeezed the sheared cord. An observational cohort of preterm infant < 35 weeks of gestation suggested that C-UCM neither improved hemoglobin levels nor reduced neonatal morbidities when compared with ICC (50). If C-UCM can be an effective method of placental transfusion of premature infants remains to be further explored.

## Complications of placental transfusion

Although there is a high demand for placental blood transfusion in preterm infants, who are expected to benefit most from this technique, some researchers still have concerns about placental transfusion for premature babies in some clinical settings. Researchers worry that the placental transfusion can lead to hyperviscosity syndrome, or polycythemia. Case-controlled trial shows that blood viscosity is related to gestational age, and all subjects’ viscosity was lower than 12P which was the diagnostic threshold for hyperviscosity (53). Other studies have not found higher rates of polycythemia or significantly increased phototherapy needs in DCC or UCM treated newborns.

In 2019, a multi-center trial (NCT 03019367) (48) regarding placental blood transfusion was terminated early. This study was to investigate the effects of different methods of placental transfusion on intracranial hemorrhage in preterm infants less than 32 weeks of gestational age. In the midterm analysis, it was found that the risk of severe IVH was significantly higher in the subgroup of vaginal delivered extremely preterm infants (23–27 gestational weeks) receiving UCM procedure, who also had higher hemoglobin levels than DCC cases. In the UCM group, the umbilical cord was squeezed four times. Similar results were not found in other gestational age subgroups. The possible reasons are as follows. First, more than 50% of extremely preterm infants face pressure associated cerebral perfusion fluctuation, which happens in only more than 20% of very preterm infants (54). Repeated UCM in preterm lambs, equivalent to human fetuses of 26 gestational weeks, resulted in significant fluctuations in carotid arterial blood flow (55). Therefore, repeated UCM in extremely preterm infants may directly cause fluctuation of cerebral blood flow, and consequently increase the risk of IVH. Second, inflammatory mediators of fetal inflammation can pass through the blood-brain barrier, and cause an inflammatory cascade reaction of central nervous system. In this case, the germinal matrix and cerebrovascular system become vulnerable to blood flow fluctuations. Lastly, for premature infants, UCM can effectively improve hemoglobin level, blood volume, and organ perfusion, but it may have an opposite effect on the immature brain. Several large cohort studies have found that the incidence of IVH in naturally delivered premature infants is significantly higher than those of cesarean section (56, 57) and UCM increases the risk of IVH. Although this study differs from other studies in many aspects, such as experimental methods, subject selection, and details of umbilical cord and placenta management, it still suggests that future studies should be more careful in assessing benefits and risks, with the ultimate goal of facilitating the survival of preterm infants and improving long-term neurological outcomes. A summary of three strategies for placental transfusion is showed in Table 2.

**TABLE 2:** Summary of three strategies for placental transfusion.

	Definition	Benefits*		Risks	
		Preterm	Term	Preterm	Term
DCC	Clamping the cord after 30 s–3 min, or after the umbilical arteries pulsation stop	• Reduce hospital mortality • Increase peak hematocrit • Reduce the need for blood transfusion • Reduce the incidence of low Apgar score at 1 min • Reduce the incidence of IVH • Higher mean arterial blood pressure in early hours after birth • Reduce the need for inotropics to low blood pressure • No significant effect on NEC, CLD, sepsis, RDS, PDA and ROP • Reduce the incidence of anemia and increase iron reserves in infancy	• Replenish blood volume • Increases hemoglobin levels and hematocrit • Assist with maintenance of body temperature • Reduce the need for blood transfusion • Higher the ferritin levels by the age of 4 months • Better motor and social skills scores at age 4 • Assist with a smooth transformation from fetal circulation to adult circulation	• Does not increase hyperbilirubinemia requiring phototherapy and symptomatic polycythemia • Possible delayed in resuscitation in non-vigorous infants • Do not increase the risk of maternal postpartum hemorrhage	
I-UCM	Squeezed the intact cord at a ength of 20 cm for 3–4 times, extruding the blood to the newborn at a rate of about 10 cm/s	• Increases hemoglobin levels • Reduce the need for blood transfusion • No significant effect on hospital mortality, NEC, CLD, sepsis, RDS, PDA and ROP	• Similar to DCC, and advantages of I-UCM are not obvious compared with DCC	• Does not increase hyperbilirubinemia requiring phototherapy and symptomatic polycythemia • Possible delayed in resuscitation in non-vigorous infants • Increased grade III∼IV IVH in premature infants < 34 weeks • Do not increase the risk of maternal postpartum hemorrhage	
C-UCM	Clamping a long segment of cord (20–40 cm) immediately after birth and milking toward the infant	• Increases hemoglobin levels • Reduce the need for blood transfusion • Less delay in resuscitation compared with DCC	• Less delay in resuscitation compared with DCC	• Does not increase hyperbilirubinemia	• Possibly less placental transfusion than I-UCM

*Benefits were relative to immediately cord clamping. CLD, chronic lung disease; C-UCM, cut umbilical cord milking; DCC, delayed cord clamping; IVH, intraventricular hemorrhage; I-UCM, intact umbilical cord milking; NEC, necrotizing enterocolitis; PDA, patent ductus arteriosus; RDS, respiratory distress syndrome; ROP, retinopathy of prematurity.

## Conclusion

Based on recent research, the mechanisms of placental transfusion have become relatively clear, and the benefits to neonates have been widely demonstrated. ICC is a practice that disrupts the normal physiologic transition at birth, it used to be widely practiced without the support by high quality evidence. DCC is widely recommended as the most physiological method of placental transfusion, meanwhile, a growing number of studies has cast doubt on the practice of UCM in premature infants. Although there are still some difficulties in performing placental transfusion for every newborn, especially for asphyxia and infants at high risk for maternal or fetal appendages. However, neonatologists should be confident in placental transfusion technology. Developing of a protocol to standardize the selection of appropriate placental transfusion strategies for different neonatal populations is also a vital task. A series of well-designed studies are warranted to assess the effects of placental transfusion in neonates with special conditions.

## Author contributions

JL wrote the manuscript. JL, GY, QW, and XZ collected and analyzed the data. RJ revised the manuscript. All authors contributed to the article and approved the submitted version.
